# Community Pharmacists’ Experiences and Attitudes towards the Provision of Sexual and Reproductive Health Services: An International Survey

**DOI:** 10.3390/healthcare11111530

**Published:** 2023-05-24

**Authors:** Javiera Navarrete, Christine A. Hughes, Nese Yuksel, Theresa J. Schindel, Shigeo Yamamura, Tomoko Terajima, Tatta Sriboonruang, Chanthawat Patikorn, Puree Anantachoti

**Affiliations:** 1 Faculty of Pharmacy and Pharmaceutical Sciences, College of Health Sciences, University of Alberta, Edmonton, AB T6G 2H1, Canada; 2 Faculty of Pharmaceutical Sciences, Josai International University, Chiba 283-8555, Japan; 3 Faculty of Pharmaceutical Sciences, Shonan University of Medical Sciences, Kanagawa 244-0806, Japan; 4 Faculty of Pharmaceutical Sciences, Chulalongkorn University, Bangkok 10330, Thailand

**Keywords:** sexual health, reproductive health, pharmacists, community pharmacy services, survey, international cooperation

## Abstract

Access to comprehensive sexual and reproductive health (SRH) services remains a challenge worldwide. Describing community pharmacists’ SRH services in countries with different scopes of practice will aid in understanding how pharmacists view their roles and how to support them in providing needed services. A cross-sectional web-based survey was administered to pharmacists working in community pharmacies in Japan, Thailand, and Canada. The survey covered 7 SRH categories: pregnancy tests, ovulation tests, contraception, emergency contraception, sexually transmitted and blood-borne infections, maternal and perinatal health, and general sexual health. Descriptive statistics were used to analyze the data. A total of 922 eligible responses were included in the analysis (Japan = 534, Thailand = 85, and Canada = 303). Most Thai and Canadian participants reported dispensing hormonal contraceptives (Thailand = 99%, Canada = 98%) and emergency contraceptive pills (Thailand = 98%, Canada = 97%). Most Japanese participants provided patient education on barrier contraceptives for men (56%) and information on the safety of medications in pregnancy (74%) and breastfeeding (76%). The majority of participants expressed interest in additional training and expanding their roles in SRH. Sharing international experiences can guide challenges faced by the evolution of pharmacists’ practice in SRH. Providing pharmacists support could help their readiness for this role.

## 1. Introduction

Access to comprehensive sexual and reproductive health (SRH) services remains a challenge worldwide. According to the World Health Organization (WHO), 4.3 billion people of reproductive age worldwide will not have access to at least one SRH intervention in their lifetime [1]. More recently, the COVID-19 pandemic has significantly impacted the provision of these services [2,3,4,5,6,7,8,9]. Fear of accessing in-person care, medication shortage, suspended services and care programs have limited access to SRH services during the pandemic [2,3,8]. As the current global agenda includes ensuring accessible and high-quality SRH services for everyone [10], finding more options for access to these services is important.

The evolution of pharmacy practice is an international phenomenon. Development, adoption, and integration of new roles and responsibilities by pharmacists have been the trend over the past few decades [11,12,13]. Balancing professional, clinical and economic considerations and adopting new roles of pharmacists are challenges for the profession worldwide [14]. Evidence for the benefits of pharmacists’ involvement in the delivery of SRH services is growing. Pharmacists have been recognized as competent and trustworthy healthcare professionals expanding access to a wide range of SRH services [15,16]. A scoping review highlighted that pharmacists’ roles in SRH have evolved beyond traditional product-focused services, such as treatment of sexually transmitted and bloodborne infections (STBBI) and contraception prescribing [16]. Challenges with the provision of SRH services by pharmacists reported in studies include integration into the pharmacy daily workflow, remuneration, cost and reimbursement for patients, and policy regulations [16]. However, most literature is from the US and UK, and there is little research describing international contexts and identifying how pharmacists can serve those needing to access SRH services.

Several factors prevent the translation of contemporary patient-oriented pharmacy practice models globally [17,18]. One of these factors is the quality and availability of information about pharmacists’ current scope of practice [17,18]. Pharmacy practice has steadily advanced over the past few decades, but significant variations in the scope of practice have been described [19]. As there is a lack of universal practice standards across pharmacy settings and countries due to differences in healthcare systems, public health agenda, pharmacy curricula, professional regulations, continuing education, and pharmacists’ compensation for professional services [18], research highlighting these differences is crucial to understand the current state of SRH services and opportunities to influence and promote further expansion of pharmacist’ roles in this area [17,20].

Given the prominent role in communities as part of primary health care, describing community pharmacists’ SRH services in countries with a different scope of practice will aid in understanding how pharmacists view their roles, see themselves as SRH providers, and what support they need to provide SRH services. To our knowledge, there is limited information regarding pharmacists’ experiences as SRH providers in Canada [21,22,23,24], and no research has described SRH services in Thailand and Japan. This study aims to explore SRH services provided by pharmacists practicing in community pharmacies in Japan, Thailand, and Canada. The secondary objectives are to identify perceived factors influencing the delivery of services and training preferences to support role expansion in each country.

### 1.1. Study Context

#### Pharmacy Education and Scope of Practice

The general characteristics and differences among educational programs, pharmacy regulations, and scope of practice between Japan, Canada, and Thailand are highlighted in Table 1.

The number of years required to obtain a pharmacy degree is similar in these countries. In Japan, completing the extended Bachelor of Pharmacy (6 years) is mandatory to qualify for the national examination for entry to practice. Influenced by the US-Thai consortium, Thailand developed their first Doctor of Pharmacy (PharmD) program in 1999. Since 2014, a 6-year PharmD program has been compulsory for national licensure [25]. Pharmacy programs in Canada implemented the PharmD degree by 2020 [26].

The scope of practice differs in each country. In Canada, Alberta is the province with the broadest scope of practice and was the first to implement prescribing authority for pharmacists in 2007 [27]. Legislation in Alberta allows pharmacists to access information in patients’ electronic health records, administer drugs by injection, order laboratory tests, and prescribe medications, including independent prescribing (additional prescribing authorization or APA) [27,28]. As a result, pharmacists in Alberta can prescribe hormonal contraception and administer progestin-only injectable contraceptives, as well as vaccines.

Pharmacy practice in Japan is principally focused on dispensing medications and providing patient counselling [29]. However, authorities and professionals in Japan are exploring how pharmacists’ roles could address different health needs [29]. Sexual freedom, recognition of health risks, and access to SRH services are current public health concerns [30]. Pharmacists are ideally situated to enhance access to services. Currently, the scope of practice allows Japanese pharmacists to counsel and educate patients on SRH topics such as pregnancy and ovulation tests, contraception as well as dispense medications prescribed by a physician [30,31].

In the last decade, Thailand has expanded community pharmacists’ roles to meet the needs of the public. After more than 10 years since transitioning to a 6-year PharmD program [32], community pharmacists offer medication reviews, risk assessments, smoking cessation and some screening services [33,34]. As part of the Universal Health Coverage Scheme, the community pharmacy is a primary care unit of the system, and pharmacists are involved in addressing several public health concerns, including adolescent pregnancy and birth rates, STBBI incidence and treatment rates [35,36,37].

**Table 1:** General characteristics, pharmacy education, and practice in Japan, Thailand, and Canada.

	Japan	Thailand	Canada
General characteristics [38,39,40,41,42]			
Population (millions)	125.7	71.6	38.25
Licensed pharmacists	302,500	47,525	48,134
Community Pharmacies	180,415	20,516	11,554
Pharmacy education [26,35,43,44,45]			
No. of pharmacy educational programs	74	19	10
Current academic credential	BPharm	PharmD	PharmD
Length of current program (years)	6	6	4
Pre-pharmacy requirements (years)	-	-	2
First year of transition from BPharm to a PharmD program (or extended BPharm)	2003	1999	2007
Completion year of transition to an all-PharmD (or extended BPharm) program	2006	2010	2020
National examination for entry to practice	Yes	Yes	Yes
Community pharmacy scope of practice [27,28,29,33,45,46,47,48]			
Availability of open-shelf drugs at the pharmacy	No	Yes	Yes
Dispensing prescription medications	Yes	Yes	Yes
Prescribing authorization	No	No	Yes
Administration of injections	No	No	Yes
Performing assessment based on symptoms	No	Yes	Yes
Performing point-of-care testing	No	No	Yes
Patient counselling and education	Yes	Yes	Yes

BPharm, Bachelor of Science in Pharmacy; PharmD, Doctor of Pharmacy.

## 2. Materials and Methods

### 2.1. Study Design

This was a descriptive, cross-sectional international study using survey methods. Results were analyzed and reported following the Strengthening the Reporting of Observational Studies in Epidemiology (STROBE) reporting guideline for cross-sectional studies [49].

An anonymous, web-based survey was distributed electronically in Japan, Thailand, and Canada (Alberta) using REDCap. REDCap is an electronic data capture tool hosted by the Women & Children’s Health Research Institute at the University of Alberta [50,51]. The survey was directed to pharmacists working in a community setting. Initially, a screening question was used to capture this information and access the survey.

The survey was conducted from June 2020 to June 2021, with each country distributing the survey at different times. The period to collect responses was 1 to 6 months; in Thailand and Japan, the survey was open for 6 months due to slower response. Details about the strategies used are provided in Table 2.

### 2.2. Instrument

The development and content of the survey have been described previously [52,53]. The instrument was developed by the research team. A literature review informed the content, and the instrument was reviewed by experts in each country. The survey was first developed in English, then translated into Japanese and Thai, and adapted to each country’s scope of pharmacist practice. The research team members and experts from Japan and Thailand reviewed the instrument for accurate translation.

The survey covered 7 common SRH categories: pregnancy tests, ovulation tests, contraception (non-hormonal and hormonal), emergency contraception, sexually and blood-borne transmitted infections, maternal and perinatal health, and general sexual health, and included 6 sections: demographics, provision of SRH services, attitudes towards the provision of SRH services, confidence when educating patients on SRH topics, factors influencing the provision of SRH services, and additional training preferences (Supplementary File S1). The survey distributed in Alberta covered additional topics reflecting the scope of practice in the region, such as the administration of injections and prescribing. Findings related to the expanded scope of practice in Alberta [53] and attitudes and practices of Japanese pharmacists regarding reproductive health services [52] have been previously published.

### 2.3. Variables

A 5-point Likert-type scale was used to assess attitudes towards (strongly disagree, disagree, neutral, agree, strongly agree) and factors influencing the provision of SRH services (no impact on the services, no effect on the services, little impact on the services, neutral, somewhat impacts the services, impacts the services to a great extent). The primary outcomes were the proportion of participants providing SRH services, the proportion of participants agreeing (or disagreeing) with a series of statements regarding pharmacists as SRH providers and the influence of factors in the provision of SRH services. Secondary outcomes were the proportion of participants interested in expanding their roles in SRH and additional training preferences, including SRH knowledge and competencies.

### 2.4. Bias

Survey responses were anonymous. A neutral language was used when designing the survey questions to minimize response bias. Increasing the number of days the survey was open and sending invitation and reminder emails were strategies used to reduce non-response bias and improve response rates [54,55,56].

### 2.5. Sample Size

There was no sample size set a priori.

### 2.6. Data Analysis

For analysis, eligibility was defined as submitted survey responses. Partial and unsubmitted responses were not included. Descriptive statistics were used to analyze the variables. Analysis was performed using Microsoft® Excel v.16.70 (Microsoft Corporation 2018).

### 2.7. Ethical Approval

This study received approval from the Josai International University Research Ethics Review Committee (10M200001), the Chulalongkorn University Research Ethics Review Committee (114.1/63), and the University of Alberta Health Research Ethics Board (Pro00095881).

## 3. Results

### 3.1. Participants

A total of 1,265 pharmacists attempted the survey (Japan = 743, Thailand = 121, Canada = 401), and 922 eligible responses from community pharmacists were included in the analysis (Japan = 534, Thailand = 85, Canada = 303). Participants were primarily female and between 20 and 40 years of age (Table 3). Regarding education and experience, most participants had a Bachelor of Pharmacy degree (Japan = 94%, Thailand = 55%, Canada = 79%). In Japan and Canada, most worked in a corporate/chain pharmacy (61% and 57%, respectively), whereas 79% worked in an independent pharmacy in Thailand.

### 3.2. Provision of SRH Services

All categories of SRH services were, to some extent, provided in the three countries (Table 4). However, differences in involvement with the provision of these services were noted. In general, most participants from Thailand and Canada provided the services captured in the survey. Most of these participants reported dispensing combined hormonal contraceptives (Thailand = 99%, Canada = 98%) and emergency contraceptive pills (Thailand = 98%, Canada = 97%). Education on combined hormonal contraceptive methods, STBBI treatment, and maternal and perinatal health were also highly reported services (Thailand = 93–99%, Canada = 82–98%). Most Japanese participants provided patient education on barrier contraceptives for men (56%) and information on the safety of medications in pregnancy (74%) and breastfeeding (76%). A smaller number of Japanese participants provided and educated on emergency contraceptive pills (7% and 15%, respectively), educated patients on STBBI prevention (13%) and treatment (18%), and eighteen percent addressed the sexual health needs of lesbian, gay, bisexual, transgender, queer or questioning, intersex, asexual, and more (LGBTQ+) individuals.

### 3.3. Attitudes towards the Provision of SRH Professional Pharmacy Services

**Figure 1:**
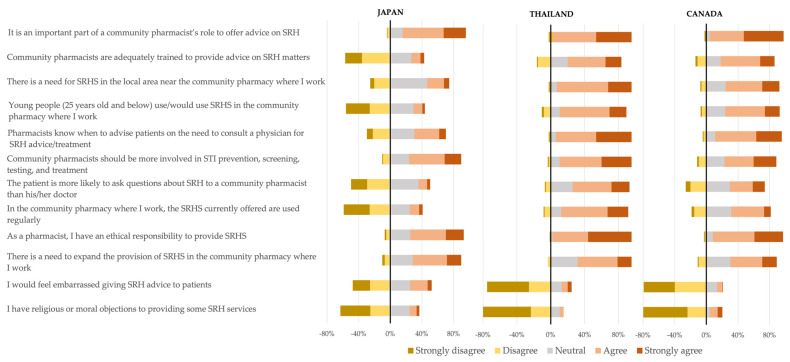
Comparison of attitudes towards the provision of sexual and reproductive health professional pharmacy services. (SRHS = Sexual and reproductive health services).

Overall, participants’ attitudes in all three countries were positive toward pharmacists’ roles in SRH (Figure 1). Most participants strongly agreed or agreed that offering advice on SRH is an essential part of community pharmacists’ roles (Japan = 80%, Thailand = 95%, Canada = 93%) and that they had an ethical responsibility as pharmacists to provide SRH services (Japan = 67%, Thailand = 97%, Canada = 89%). Japanese participants were more likely to strongly disagree or disagree with statements regarding the need for SRH services in their local area (25%) and the use of SRH services by young people (56%). Sixty-four percent of Thai participants and 68% of Canadian participants believed pharmacists are adequately trained to provide SRH services, while over half of Japanese participants disagreed with this statement.

### 3.4. Perceived Factors Influencing the Provision of SRH Professional Pharmacy Services

**Figure 2:**
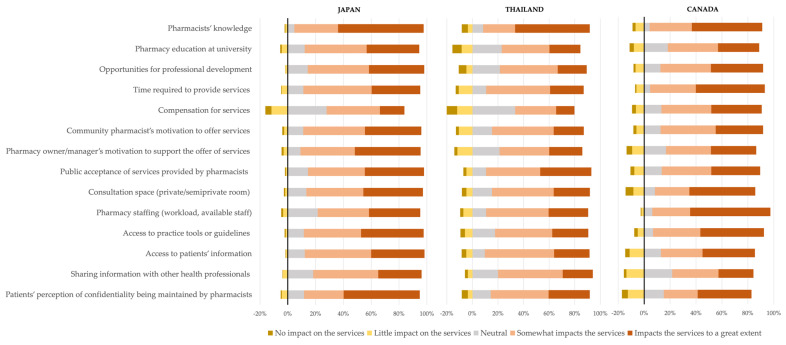
Comparison of perceived factors influencing the provision of sexual and reproductive health professional pharmacy services.

In general, most participants from the three countries responded that all factors included in the survey influenced the provision of SRH services to some extent (Figure 2). In Japan, 93% of participants indicated pharmacists’ knowledge “somewhat impacts the service” or “impacts the services to a great extent.” Similarly, most Thai and Canadian participants highlighted the influence of pharmacists’ knowledge (83% and 86% respectively). A higher percentage of all Canadian participants indicated pharmacy staffing (91%) influenced the provision of these services to a great extent, as compared to Japanese (74%) and Thai participants (80%). Compensation for the service “somewhat impact the service” or “impact the services to a great extent” for about half of participants in Japan and Thailand (56% and 46%, respectively), whereas for Canadian participants compensation had more impact on the provision of SRH services (77%).

### 3.5. Preferences for Additional Training on SRH

Most respondents expressed interest in additional training in SRH services (Japan = 79%, Thailand = 91%, Canada = 84%). More than half of the participants in Japan (54%) and about three-quarters of the participants in Thailand and Canada (77% and 71%, respectively) were interested in expanding their roles in SRH.

**Figure 3:**
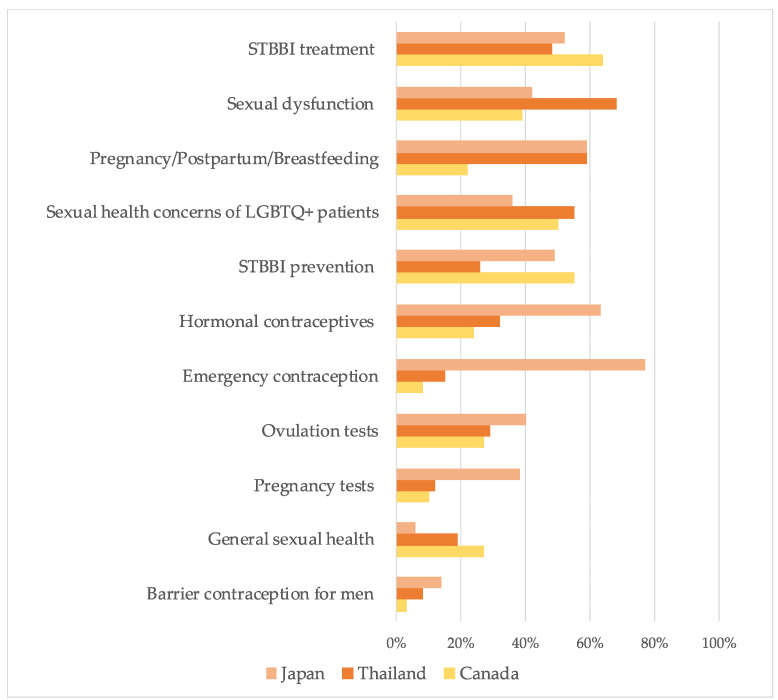
Comparison of preferences for additional training on sexual and reproductive health topics.

Participants’ preferences varied between countries. For Japanese participants interested in additional training, the top preferences were emergency contraception (77%) and hormonal contraceptives (63%), followed by pregnancy/postpartum/breastfeeding (59%). Additionally, the preferred areas for Thai participants were sexual dysfunction (68%), pregnancy/postpartum/breastfeeding (59%), and sexual health concerns of LGBTQ+ patients (55%). In Canada, approximately half of the participants or more preferred more training in the areas of STBBI treatment (64%), STBBI prevention (55%), and sexual health concerns of LGBTQ+ patients (50%) (Figure 3).

**Figure 4:**
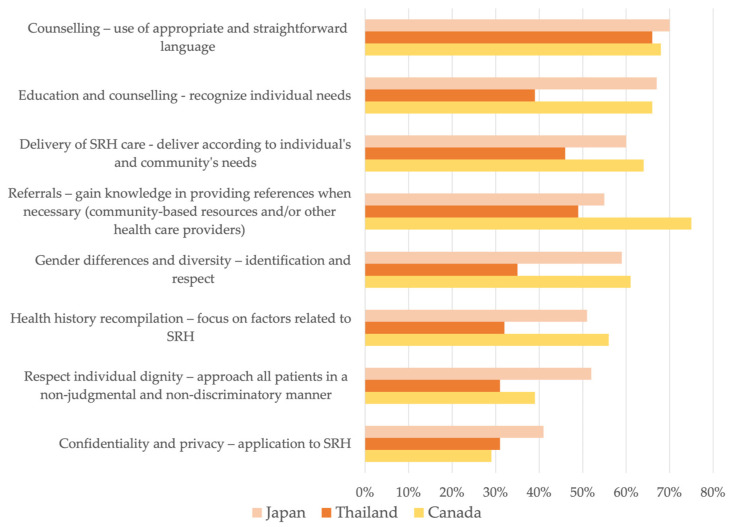
Comparison of training interests in sexual and reproductive health competencies.

In terms of SRH competencies, more than 60% reported interest in additional training related to the use of appropriate and straightforward language when counselling patients (Japan = 70%, Thailand = 66%, Canada = 60%). This was the preferred choice for participants in Japan and Thailand, while for Canadian participants, their top selection was additional training on providing referrals (75%). Less than half of the participants reported interest in additional training related to confidentiality and privacy (Japan = 41%, Thailand = 31%, Canada = 29%). A higher proportion of Japanese participants considered approaching individuals nonjudgmentally a relevant topic for training (55%) (Figure 4).

## 4. Discussion

This multi-country study examined SRH services provided by pharmacists from regions with different regulations around pharmacy practice and perspectives regarding SRH. Results showed that pharmacists were involved in providing SRH services and had a positive attitude towards pharmacists’ roles in SRH. Participants’ perceptions of factors influencing the provision of SRH were similar in the three countries, as well as the willingness to expand roles and receive additional SRH training. The findings from this study can be used to inform education and training needs as well as potential barriers to the adoption of SRH services offered by community pharmacists.

Overall, a high percentage of Alberta pharmacists reported providing patient education across several SRH areas in addition to the provision of emergency contraception. This may be a reflection of changes in the scope of practice [57] and health policy and regulations in Alberta, Canada. In Thailand, pharmacy practice has evolved as well, and SRH services have been recognized as a pharmacy service [33]. Thai participants were highly involved with patient education in SRH, including emergency contraception and STBBI treatment and prevention. In Japan, the evolution of pharmacy practice has been more gradual compared to the other two countries [29,45]. In addition, sexual freedom, recognition of health risks, and access to SRH services are more recent public health concerns [31,58,59,60]. Overall, a lower percentage of Japanese participants provided SRH services included in this survey, including emergency contraception. Participants were more likely to report giving information related to the safety of medications in pregnancy and breastfeeding.

We found that community pharmacists from countries with different scopes of practice see themselves as SRH providers and consider offering advice on SRH topics an essential part of their roles. Still, they face similar challenges when providing SRH services. Regardless of differences in educational, economic, and healthcare system contexts, a set of competencies essential for patient care practice are similar in all regions [17,61]. Provision of SRH services, along with professional ethics and collaboration with patients and other health care providers, align with participants’ views to meet patients’ needs as SRH providers. All factors included in this survey were reported to influence the provision of SRH services to some extent. Knowledge of SRH, pharmacy staffing, and pharmacists’ compensation were some of the main influencing factors for participants. Previous studies have identified barriers to implementing pharmacy services, including pharmacists’ knowledge and the time required to provide the services [16,62].

One of the common findings was the willingness to receive more SRH training. However, the top preferences differed between countries, which can be influenced by the role participants associate with being an SRH provider. The focus of Japanese participants was emergency contraception, one of the main SRH discussion topics on the public agenda in Japan [30]. It is not surprising that Japanese pharmacists are interested in learning more about emergency contraception as their responsibilities have changed recently and will continue to change [52]. Alberta participants’ primary preference was STBBI treatment. A syphilis outbreak was declared in 2019 [63]. Incidence rates of other sexually transmitted infections (chlamydia, gonorrhea) have continued to increase [64]. At the same time, the incidence of the human immunodeficiency virus (HIV) and hepatitis C virus (HCV) have not declined despite the availability of treatment and prevention tools [65]. In Thailand, sexual dysfunction was the most preferred topic for additional training. The high demand for medicines for sexual dysfunction [66,67], the challenges of their distribution (e.g., sildenafil is largely misused and is one of the most counterfeited drugs worldwide) [68], and the widespread use of herbal products with no scientific evidence for sexual health dysfunction may explain the preference of Thai participants.

To position pharmacists as SRH providers and increase access to SRH services, it is relevant that they see themselves as SRH providers. Recognizing that pharmacists’ process to identify as SRH providers is dynamic, complex, and influenced by many factors will help to find the best tools and strategies to support their identity formation. Sharing international experiences on the influence of the changes in the profession can help to face challenges brought by this process. Exploring what different countries do to support their pharmacy workforce to prepare them to be SRH providers can inform the development of training programs and implementation strategies customized to the practice environment. Expanding the offer of continuing education programs and creating pharmacist-specific guidelines and practice tools based on the context of each jurisdiction are some of the recommendations of this work.

### Strengths and Limitations

The strengths of this study include the description of community pharmacists’ perspectives regarding the provision of SRH services in Canada, Japan, and Thailand. This study is the first to characterize perceptions of SRH practice in several topics from pharmacists in countries with different scopes of practice. Despite these strengths, several limitations need to be considered. This study may not be generalized to other regions in the selected countries and nations. Due to our study design, the results are limited due to volunteer, acquiescence, social desirability, and non-response bias. The sample size was not calculated a priori, and the number of participants varied between countries. No current detailed pharmacy workforce statistics are available to consider the representativeness of the population we aimed to survey in Japan and Thailand. However, the representativeness of this sample of the Canadian (Alberta) pharmacists’ population has been described before [53]. The survey was developed and distributed during the COVID-19 pandemic, which impacted the distribution and recruitment processes, resulting in slightly different methods and timelines used in the three countries. In addition, the global health crisis may have affected pharmacists’ workload and impacted our response rates.

## 5. Conclusions

Community pharmacists in countries with different scopes of practice and pharmacy regulations reported providing services in several SRH areas. While the extent to which pharmacists self-report the provision of SRH services varied in each country, pharmacists were interested in expanding SRH services and additional SRH training. Addressing factors that influence the provision of SRH services and supporting pharmacists is essential for expanding access. Sharing international experiences can guide challenges faced with the evolution of practice in SRH. Introducing support strategies based on local needs and international experiences, such as creating continuing education opportunities and practice tools, could help support pharmacists in providing care for individuals seeking SRH services.

## Figures and Tables

**Table 2:** Procedures for data collection.

	Japan	Thailand	Canada
Recruitment strategy	Emails were sent to contact lists from pharmacy professional organizations, pharmacy and drugstore chains, and community pharmacist groups. Attendees of continuing education conferences. Facebook and Twitter promotion [52].	Emails sent to contact list from regional professional pharmacy associations	Emails sent to contact list from the regional professional regulatory body (Alberta)
Distribution	Email	Email	Email
Total number of pharmacists contacted	N/A	760 *	5349 *
Responses	534	85	303
Estimated response rate	N/A	N/A	8% [53]
Reminders	None	3	3
Dates of data collection	November 2020– April 2021	December 2020– June 2021	June 2020– July 2020
Duration of open survey	6 months	6 months	2 months
Incentive	None	None	$100 gift card draw (odds of winning 1 in 10)

* Total number of people contacted. This number includes pharmacists working in different settings, not only community pharmacy. N/A, not available.

**Table 3:** Participants characteristics.

Characteristics	Japan (N = 534) *n* (%)	Thailand (N = 85) *n* (%)	Canada (N = 303) *n* (%)
Gender			
Female	295 (55)	54 (64)	199 (66)
Age range			
20–30 years	208 (39)	17 (20)	71 (24)
31–40 years	116 (22)	40 (47)	120 (40)
41–50 years	97 (18)	11 (13)	60 (20)
51–60 years	81 (15)	8 (9)	41 (14)
61–70 years	29 (5)	7 (8)	9 (3)
71+ years	3 (1)	2 (2)	0 (0)
Professional Education			
Bachelor of Pharmacy (4 years)	245 (46)	47 (55)	239 (79)
Bachelor of Pharmacy (6 years)	257 (48)	-	-
Doctor of Pharmacy (PharmD)	-	21 (25)	32 (11)
Residency	-	-	3 (1)
Post-professional or post-baccalaureate PharmD	-	-	3 (1)
Master (M.Sc. or M.Pharm.)	23 (4)	12 (14)	20 (7)
Doctor of Philosophy (Ph.D.)	6 (1)	2 (2)	4 (1)
Years of registration as a pharmacist			
<1 year	9 (2)	2 (2)	16 (5)
1–5 years	214 (40)	16 (19)	92 (30)
6–10 years	50 (9)	21 (25)	63 (21)
11–20 years	91 (17)	23 (27)	68 (22)
21–30 years	95 (18)	8 (9)	38 (13)
>31 years	74 (14)	15 (18)	25 (8)
Type of pharmacy			
Independent	68 (13)	67 (79)	83 (27)
Corporate/chain	322 (61)	12 (14)	172 (57)
Banner/franchise	1 (0)	6 (7)	44 (15)
Drugstore chain	135 (26)	-	-
Other	4 (1)	0 (0)	3 (1)

**Table 4:** Sexual and reproductive health services provided by community pharmacists.

Service	Country		
	Japan (N = 534) *n* (%)	Thailand (N = 85) *n* (%)	Canada (N = 303) *n* (%)
Pregnancy Tests			
Patient education on pregnancy tests	192 (36)	83 (98)	211 (70)
2. Ovulation Tests			
Patient education on ovulation tests	222 (42)	70 (83)	152 (50)
3. Contraception			
Provide			
Combined hormonal contraceptives	250 (47)	84 (99)	298 (98)
Patient education on			
Combined hormonal contraceptives	207 (39)	84 (99)	295 (97)
Barrier contraception for men	299 (56)	67 (79)	210 (69)
4. Emergency contraception			
Provision of EC pills (Levonorgestrel only pills, e.g., Plan B^®^)	37 (7)	82 (98)	294 (97)
Patient education on EC (Levonorgestrel only pills, e.g., Plan B^®^)	81 (15)	84 (99)	296 (98)
5. STBBI (chlamydia, gonorrhea, hepatitis B and C, genital herpes, syphilis, trichomoniasis, HIV, HPV)			
Patient education on STBBI treatment	96 (18)	82 (97)	247 (82)
Patient education on STBBI prevention	68 (13)	80 (94)	216 (71)
6. Maternal and perinatal health			
Patient education on nutrition and vitamin supplementation for prenatal and pregnancy care	253 (48)	79 (93)	278 (92)
Provision of information on safety of medications in pregnancy	395 (74)	85 (100)	297 (98)
Provision of information of recommended vaccines prior to and during pregnancy	139 (26)	39 (46)	259 (86)
Provision of information on safety of medications in breastfeeding	408 (76)	85 (100)	298 (98)
7. General Sexual Health			
Patient education on sexual dysfunction related to medications	159 (30)	51 (61)	255 (84)
Assist female patients identify/select options for sexual dysfunction	61 (11)	33 (39)	102 (34)
Assist male patients identify/select options for sexual dysfunction	67 (13)	53 (63)	207 (68)
Address sexual health concerns/needs of LGBTQ+ patients	95 (18)	40 (48)	132 (44)

EC, emergency contraception; STBBI, sexually transmitted and blood-borne infection; HIV, human immunodeficiency virus; HPV, human papillomavirus; LGBTQ+, lesbian, gay, bisexual, transgender, and queer (or questioning) and others.

## Data Availability

The data presented in this study are available on request from the corresponding author.

## Supplementary Materials

The following supporting information can be downloaded at: https://www.mdpi.com/article/10.3390/healthcare11111530/s1, File S1: SRH survey.
