# Supplementary material for: Community Pharmacists’ Experiences and Attitudes towards the Provision of Sexual and Reproductive Health Services: An International Survey
Source: Healthcare (Basel). 2023 May 24;11(11):1530. doi: 10.3390/healthcare11111530 (PMC10252229; doi:10.3390/healthcare11111530)
Supplement: Supplementary file 1 [file healthcare-11-01530-s001.zip › healthcare-2390474-supplementary.pdf]

International comparison of community pharmacists' roles in provision of Sexual and Reproductive Health (SRH) services

Demographics

1. What is your age range?
   - o 20-30 years
   - o 31-40 years
   - o 41-50 years
   - o 51-60 years
   - o 60-71 years
   - o 70+ years

2. Please indicate your gender
   - o Male
   - o Female
   - o Non-binary
   - o Prefer not to answer

3. How many years of licensure do you have?
   - o < 1 year
   - o 1-5 years
   - o 6-10 years
   - o 11-20 years
   - o 21-30 years
   - o >31 years

4. What is your highest level of education related to pharmacy?
   - o BSc in pharmacy
     - a. 4-years *(Japan only)*
     - b. 6-years *(Japan only)*
   - o Enter-to-practice PharmD
   - o Residency *(Canada only)*
   - o Post-professional or post-baccalaureate PharmD *(Canada only)*
   - o Master's degree (M.Sc. or MPharm)
   - o Ph.D.
   - o Other (please specify): ____

5. What is your current position status? Please select all that apply.
   - o Staff Pharmacist (e.g. full time, part time, permanent, etc.)
   - o Relief/Casual Pharmacist
   - o Pharmacy Owner
   - o Pharmacy Manager
   - o Pharmacy Consultant
   - o Other (please specify): ____________________

6. What are the areas in which you primarily practice? Please select all that apply.
   - o Community pharmacy- independent
   - o Community pharmacy- corporate/chain
   - o Community pharmacy- banner/franchise
   - o Drugstore chain *(Japan only)*
   - o Other (please specify):________________

I. Provision of Sexual and Reproductive Health (SRH) Services

Please indicate whether the following products and services are currently provided at the pharmacy where you work as well as plans to provide these products/services in the future.

	Currently provided?		Plan to provide in future?		
	YES	NO	YES	NO	DO NOT KNOW
**A. Pregnancy Tests**					
A1. Do you provide patient education on pregnancy tests?	○	○	○	○	○
**B. Ovulation Tests**					
B1. Do you provide patient education on ovulation tests?	○	○	○	○	○
**C. Contraception**					
C1. Do you provide patient education on male non-hormonal (barrier) contraceptives?	○	○	○	○	○
C2. Do you provide combined hormonal contraceptives (CHC)?	○	○	○	○	○
C3. Do you provide patient education on hormonal contraception?	○	○	○	○	○
**E. Emergency contraception**					
E1. Do you provide emergency contraception pills (ECPs)?	○	○	○	○	○
E2. Do you provide patient education on ECPs?	○	○	○	○	○
**F. Sexually Transmitted Infections (STIs)**					
F1. Do you provide patient education on STIs treatment?	○	○	○	○	○
F2. Do you provide patient education on preventing STIs?	○	○	○	○	○
**G. Maternal and perinatal health**					
G1. Do you provide patient education on nutrition and vitamin supplementation for prenatal and pregnancy?	○	○	○	○	○
G2. Do you provide information on safety of medications in pregnancy?	○	○	○	○	○
G3. Do you promote recommended vaccines for women prior to and during pregnancy?	○	○	○	○	○
G5. Do you provide information on safety of medications in breastfeeding?	○	○	○	○	○
**H. General Sexual Health**					

LGTBQ+: Lesbian, gay, bisexual, transgender and queer (or questioning) and others.

H1. Do you provide patient education on sexual dysfunction related to medications?	○	○	○	○	○
H2. Do you assist female patients on options for sexual dysfunction?	○	○	○	○	○
H3. Do you assist male patients on options for sexual dysfunction?	○	○	○	○	○
H4. Do you address health concerns/needs of LGBTQ+ patients?	○	○	○	○	○

LGTBQ+: Lesbian, gay, bisexual, transgender and queer (or questioning) and others.

II. Attitudes toward Sexual and Reproductive Health (SRH) services

Please indicate how strongly you agree or disagree with each of the following statements about SRH services:

	Strongly disagree	Disagree	Neutral	Agree	Strongly agree
a. It is an important part of a community pharmacist's role to offer advice on sexual and reproductive health	○	○	○	○	○
b. Community pharmacists are adequately trained to provide advice on sexual and reproductive health matters	○	○	○	○	○
c. There is a need for sexual health and reproductive services in the local area near the community pharmacy where I work	○	○	○	○	○
d. Young people (25 years old and below) use/would use sexual and reproductive health services in the community pharmacy where I work	○	○	○	○	○
e. Pharmacists know when to advise patients on the need to consult a physician for sexual and reproductive health advice/treatment	○	○	○	○	○
f. Community pharmacists should be more involved in sexually transmitted infections prevention, screening, testing, and treatment	○	○	○	○	○
g. The patient is more likely to ask questions about sexual and reproductive health to a community pharmacist than his/her doctor	○	○	○	○	○
h. I would be embarrassed giving sexual and reproductive health advice to patients	○	○	○	○	○
i. I have religious or moral objections to providing sexual and reproductive health services	○	○	○	○	○
j. In the community pharmacy where I work, the sexual and reproductive health services currently offered are used regularly	○	○	○	○	○
k. As a pharmacist, I have an ethical responsibility to provide SRH services	○	○	○	○	○
l. There is a need to expand the provision of sexual and reproductive health services in the community pharmacy where I work	○	○	○	○	○

Is there any **belief related to the provision of SRH services** that you would like to tell us about?
____

LGTBQ+: Lesbian, gay, bisexual, transgender and queer (or questioning) and others.

III. Factors that influence community pharmacy SRH services

Please indicate to what extent you think the following factors impact the provision of sexual and reproductive health services at the community pharmacy where you work?

	No impact on the services	Little impact on the services	Neutral	Somewhat impacts the services	Impacts the services to a great extent
a. Pharmacists' knowledge	○	○	○	○	○
b. Pharmacy education at university	○	○	○	○	○
c. Opportunities for professional development	○	○	○	○	○
d. Time required to provide services	○	○	○	○	○
e. Compensation for services	○	○	○	○	○
f. Community pharmacist's motivation to offer services	○	○	○	○	○
g. Pharmacy owner/manager's motivation to support the offer of services	○	○	○	○	○
h. Public acceptance of services provided by pharmacists	○	○	○	○	○
i. Consultation space (private/semiprivate room)	○	○	○	○	○
j. Pharmacy staffing (workload, available staff)	○	○	○	○	○
k. Access to practice tools or guidelines	○	○	○	○	○
l. Access to patients' information	○	○	○	○	○
m. Sharing information with other health professionals	○	○	○	○	○
n. Patients' perception of confidentiality being maintained by pharmacists	○	○	○	○	○

Please provide any additional comments you might have about factors that influence the provision of SRH services below.

____

IV. Sexual and Reproductive Health (SRH) competencies and training preferences

1. Would you like to expand your role in SRH services?

YES NO

O O

2. Would additional training be beneficial in expanding your role in SRH services?

YES NO

O O

LGTBQ+: Lesbian, gay, bisexual, transgender and queer (or questioning) and others.

3. Please select the TOP 5 (five) SRH topics **you would like to receive more training**:

- ○ Pregnancy tests
- ○ Ovulation tests
- ○ Barrier contraception for men (e.g. condoms)
- ○ Hormonal contraception
- ○ Emergency contraception
- ○ Sexually transmitted and blood-borne infections (STBBI) treatment
- ○ STBBIs prevention
- ○ Pregnancy/Postpartum/Breastfeeding
- ○ Sexual dysfunction
- ○ Sexual health concerns of LGBTQ+ patients
- ○ General Sexual Health
- ○ None of above

4. The World Health Organization (WHO) identifies core SRH competencies that are desirable to deliver SRH services. These competencies are useful to protect, provide and promote SRH in the community.
From the following competencies suggested by the WHO, **please select the ones you would be interested in having more training: (select all that apply)**

SRH skills, attitudes, and knowledge

- o Respect individual dignity – approach all patients in a non-judgmental and non-discriminatory manner
- o Confidentiality and privacy – application to SRH
- o Gender differences and diversity – identification and respect
- o Education and counselling - recognize individual needs
- o Counselling – use of appropriate and straightforward language
- o Delivery of SRH care - deliver according to individual's and community's needs
- o Health history recompilation – focus on factors related to SRH
- o Referrals – gain knowledge in providing references when necessary (community-based resources and/or other health care providers)

LGTBQ+: Lesbian, gay, bisexual, transgender and queer (or questioning) and others.
